# Species-specific SNP arrays for non-invasive genetic monitoring of a vulnerable bat

**DOI:** 10.1038/s41598-024-51461-5

**Published:** 2024-01-22

**Authors:** Rujiporn Thavornkanlapachai, Kyle N. Armstrong, Chris Knuckey, Bart Huntley, Nicola Hanrahan, Kym Ottewell

**Affiliations:** 1 Department of Biodiversity, Conservation and Attractions, Bentley Delivery Centre, Locked Bag 104, Bentley, WA 6983 Australia; 2 School of Biological Sciences, The University of Adelaide, Adelaide, SA 5005 Australia; 3 South Australian Museum, Adelaide, SA 5000 Australia; 4 Biologic Environmental, 24 Wickham Street, East Perth, WA 6004 Australia; 5 Hawkesbury Institute for the Environment, Western Sydney University, Richmond, NSW 2753 Australia; 6 Research Institute for the Environment and Livelihoods, Charles Darwin University, Darwin, NT 0815 Australia

**Keywords:** Genotyping and haplotyping, Ecology

## Abstract

Genetic tagging from scats is one of the minimally invasive sampling (MIS) monitoring approaches commonly used to guide management decisions and evaluate conservation efforts. Microsatellite markers have traditionally been used but are prone to genotyping errors. Here, we present a novel method for individual identification in the Threatened ghost bat *Macroderma gigas* using custom-designed Single Nucleotide Polymorphism (SNP) arrays on the MassARRAY system. We identified 611 informative SNPs from DArTseq data from which three SNP panels (44–50 SNPs per panel) were designed. We applied SNP genotyping and molecular sexing to 209 *M. gigas* scats collected from seven caves in the Pilbara, Western Australia, employing a two-step genotyping protocol and identifying unique genotypes using a custom-made R package, ScatMatch. Following data cleaning, the average amplification rate was 0.90 ± 0.01 and SNP genotyping errors were low (allelic dropout 0.003 ± 0.000) allowing clustering of scats based on one or fewer allelic mismatches. We identified 19 unique bats (9 confirmed/likely males and 10 confirmed/likely females) from a maternity and multiple transitory roosts, with two male bats detected using roosts, 9 km and 47 m apart. The accuracy of our SNP panels enabled a high level of confidence in the identification of individual bats. Targeted SNP genotyping is a valuable tool for monitoring and tracking of non-model species through a minimally invasive sampling approach.

## Introduction

Species worldwide are experiencing dramatic population declines leading to extirpation and extinction, which has largely been attributed to human activities^1^. To understand threatening processes, implement conservation actions and evaluate outcomes, wildlife monitoring has traditionally employed invasive live capture techniques which can inflict stress, injuries or even death to the animals that are being studied^2^. Traditional marking and sampling practices, including catching, toe clipping, physical tagging, attaching data loggers, and taking blood and tissue samples, can negatively affect animals’ physiology and behaviour^2^. There have been calls for more compassionate wildlife research to investigate and implement less invasive monitoring methods^2^. Minimally invasive or non-invasive (MIS) monitoring consists of methods to monitor animals without disturbance to their normal behaviour, ecology or physiology. Genetic monitoring using non-invasively collected samples such as hairs, faeces, feathers, shells and shed skin has been implemented for many vertebrate species^3–5^. With the advancement of genetic sequencing platforms and improved DNA extraction methods, MIS genetic monitoring offers the ability to monitor population demography (e.g. population size and trajectory^6^), as well as gain insight into microevolutionary processes such as, genetic diversity^7^, hybridization^8^, movement, dispersal and migration^6,7,9^ and social structure^10^, as well as assisting in identification of genetic stocks for management^11^ and diet analysis^12^.

Whilst minimally invasive sampling can alleviate disruption and harm to individuals, its application in wildlife monitoring is not without challenges. Scats are easily located for many species and are the basis for one of the most frequently used MIS methods, however, the quality and quantity of DNA obtained from scats are dependent on scat age, environmental conditions, and the concentration of digestive by-products derived from the target species’ diet. Rapid degradation of DNA typically occurs in the first 5–7 days post-deposition^13,14^ but, in some species, it can occur at the slower rate of 2–3 weeks^4,5^. Environmental conditions such as exposure to sunlight, high temperature, and precipitation can accelerate degradation rate, decrease genotyping success, and inflate genotyping error rate^4,5,15–17^. In addition, PCR inhibition by digestive by-products, DNA extraction by-products or DNA preservatives can all lower amplification success^18^.

Faecal genotyping with microsatellite markers has been applied successfully in wildlife monitoring for many years^5,15,16^. The benefit of microsatellite markers is that statistical power to identify individuals is achieved with relatively few markers but many alleles. However, for low and/or poor-quality DNA samples, laboratory artefacts such as stutter peaks, false alleles and allelic dropout can reduce the reliability of genotype calling. While this can be ameliorated by applying additional laboratory protocols (e.g., multiple PCR replicates and calling a consensus genotype) and manually assessing genotypes to ensure correct and consistent calls, both are expensive and time-consuming^19,20^. Moreover, comparing results generated by different laboratories and/or laboratory technicians requires further standardising to maintain accuracy, making multi-year or multi-jurisdictional projects difficult. Recent advances in DNA sequencing technology have led to the expanded use of single-nucleotide polymorphism (SNP) markers in wildlife genetic studies. Unlike microsatellites, SNPs have fewer alleles per locus–theoretically up to four, but mostly two due to low mutation rates^21^; and larger numbers of loci can be surveyed simultaneously to achieve similar resolution as microsatellite markers. A panel of moderately polymorphic ~ 100 SNPs (minimum allele frequency of 0.2) is sufficient to provide statistical power to estimate pedigree relationships equivalent to 16–20 microsatellites, and less SNPs required if they are highly polymorphic^21^. Species-specific SNPs can be identified, and due to their binary nature, SNPs are relatively easy to score, analyse and compare between laboratories. SNP markers have been shown to outperform microsatellite markers for faecal DNA genotyping with higher precision, lower genotyping error rates and requiring fewer repeats^22^. Once a suite of informative markers is identified, they can be pre-selected into SNP panels allowing automated genotyping on array-based platforms such as MassARRAY, Ampliflour and Fluidigm; see^3^.

The ghost bat (*Macroderma gigas*) is one of the largest microchiropteran bats in the world^23^, with the largest individuals weighing 140–165 g and a wingspan of 60 cm^23,24^. The species has a slow reproductive rate, with females breeding from 2 to 3 years of age^25^ with a generation time of 8 years^26^. *M. gigas* was once distributed widely over most of Australia (excluding Tasmania and Victoria^27^) but their distribution has now contracted to several geographic isolates across northern Australia in response to increasing aridity and anthropogenic threats^26^. Key identified threats include the loss of roost sites and foraging habitat from mining, the disturbance of roosts during biological surveys, the deterioration in condition and flooding within old underground mines used for roosting, entanglement in barbed wire fences, foraging habitat modification for livestock, cave tourism, competition with introduced foxes and cats and poisoning by ingestion of cane toads^26,28,29^. The Pilbara population is the most geographically and genetically isolated population of *M. gigas*^26,30^. It is estimated to consist of ~ 1200 bats^31^ while global population is estimated to be < 10,000^23,26^. Most known large colonies are located in present and past mining exploration areas^23^. Australia-wide, it is estimated that the global population of *M. gigas* has declined between 16–45%^23^ and the species is currently listed as Vulnerable on the International Union for Conservation of Nature (IUCN) Red List of Threatened Species^28^, and on the Australian Commonwealth Government *Environment Protection and Biodiversity Conservation Act 1999*.

Monitoring demographic processes and assessing trends in population size of this species have been particularly challenging. Rather than capturing bats, colonies of *M. gigas* are most often monitored through visual counts of individuals within roosts or exiting roosts after sunset or by quantifying bat activity from social and echolocation calls on bat detector recordings^32^. In general, Capture-Mark-Recapture (CMR) methodologies can provide additional information on bat populations, where recaptures over multiple CMR sessions can be used to track the movements of individuals; and the identification of novel captured individuals allows population size, longevity and recruitment within a population to be monitored. CMR relies on being able to recapture and identify individuals, which is achieved typically through live capture and by tagging using radio-frequency identification (RFID) or other methods of marking^33–35^. However, CMR analyses from these methods can be unreliable due to heterogeneity in individual detection probability, low capture/recapture rates, trap shyness, or tag loss^36^. The use of ‘molecular tags’, i.e., using individual genotypes generated from scats, can overcome many of these issues because tags are ‘permanent’ and capture/recapture does not rely on the target species encountering and entering traps. Thus, molecular tagging represents a robust alternative to the CMR method to monitor *M. gigas* colonies, and with minimal interference as an additional advantage.

Scat genotyping has been applied successfully in previous studies of *M. gigas* using microsatellite markers^37–39^, but due to the high genotyping error rate, can lead to uncertain identification of captured/recaptured bats and consequently inaccurate estimation of population size. SNP panels offer improved reliability and accuracy of genotyping^18^, and enable pre-selection of highly informative markers with additional statistical power to discriminate between highly-related individuals. Here, we describe the development of a set of 140 novel SNP markers designed specifically for application on the MassARRAY genotyping platform, plus a modification of four sexing markers from Ottewell et al.^38^, to facilitate rapid genotyping for *M. gigas* monitoring in the Pilbara region of Western Australia. We began with SNP marker selection, SNP panel design and in silico evaluation of marker informativeness. We then evaluated the performance of SNP panels and modified sexing markers in a case study of *M. gigas* roosts at West Angelas, in the eastern Hamersley Range in the Pilbara, and assessed the application of these markers in identifying temporal and spatial cave usage of *M. gigas* in the study area.

## Materials and methods

### Identification of SNP loci and MassARRAY SNP assay design

We obtained short read genomic data from a previous study of eight Pilbara colonies of *M. gigas* (K.N. Armstrong, unpublished data; generated by Diversity Arrays Technology Pty Ltd, Canberra). From trimmed 69 bp reads, 33,340 informative SNP loci were identified for individual and population genetic analysis using Diversity Arrays Technology (DArT) from 120 tissue samples. To identify informative and high-quality loci for individual identification, we screened SNP loci for those suitable for MassARRAY primer design (single SNP per locus, > 25 bp flanking sequence), with high read depth and coverage, high information content (heterozygosity, minor allele frequency) and removing those failing tests for Hardy–Weinberg equilibrium and linkage disequilibrium (Table 1). SNPs were filtered using custom scripts, and functions from the dartR version 1.9.6^40^ and SNPRelate version 0.9.19 packages^41^ in the R Statistical Software v4.0.3^42^. The script used to filter informative SNPs is provided in Supplementary 1. To determine the number of SNPs to be included in the MassARRAY SNP panels, we compared genetic diversity estimates and genetic structure in silico using four sets of randomly selected SNPs (n = 50, 100, 150, 200). Population genetic diversity statistics observed heterozygosity (Ho), gene diversity or expected heterozygosity (Hs), and the inbreeding coefficient (F_IS_) were calculated and permuted 500 times for sites that had sample size > 8 in the Hierfstat package version 0.5-10 using R^42^. Population structure was analysed using a Pearson Principal Component Analysis implemented in the dartR package with the gl.pcoa function^40^. Probability of identity (P_ID_) was estimated using GENALEX v6.5 to determine the number of loci needed to distinguish unique individuals^43^. We chose the threshold of 0.0001 or exclusion probability > 99.9% as suggested by Waits et al.^44^ for SNP data from natural populations.

**Table 1:** *Macroderma gigas* SNP filtering steps to select potential loci from DArTseq for MassARRAY panels.

Filter	No. of SNPs	No. of individual
Raw data	33,340	120
Loci that have only 1 SNP	18,616	120
Sequence length > = 50 base pairs	16,377	120
SNP position 25 to 45	5551	120
Average read depth per locus between 5 and 200	5179	120
Genotyping rate per locus > = 0.80	3047	120
Genotyping rate per individual > = 0.80	3043	119
The ratio of allele read depth difference between Reference and Alternative SNP 0.2–0.8	3038	119
Reproducibility > 0.95 in technical replicates	2875	119
Paralog (sequence similarity of ≥ 25% removed)	2769	119
Heterozygosity between 0.2–0.5	1273	119
Minimum allele frequency between 0.3–0.5	625	119
Select for loci in Hardy–Weinberg equilibrium	625	119
Loci in linking equilibrium	611	119

Sequence information for the filtered loci (n = 611) was sent to the Australian Genome Research Facility, Brisbane (AGRF) to identify three multiplex SNP panels of 44–49 SNPs each and design primers using the Assay Design Suite (v2.2, Agena Bioscience, San Diego, CA, USA). We trialled the performance of designed panels with seven tissue and 11 scat samples, tested the effect of DNA concentration on amplification success and error rate, and, lastly, compared the relative efficiency of two different DNA extraction kits, the Omega Biotek Mag-Bind Stool DNA 96 kit (Omega, USA, Cat No: M4016-01) and QIAamp^®^ Fast DNA Stool Mini kit (Qiagen, Germany, Cat No: 51604) using scat samples from this case study (n = 209) and from a previously published study (Ottewell et al.^38^, n = 160). SNP genotyping was carried out on the MassARRAY system (Agena Biosciences, San Diego, CA, USA) at AGRF. Amplification and extension reactions were performed with the iPLEX Gold Reagent Kit (Agena Bioscience, San Diego, CA, USA) according to the manufacturer's protocols using 1 μL of extracted faecal DNA. Resultant SNP genotypes were identified by mass spectrometry and called by AGRF using MassARRAY TyperAnalyzer 4.1 software (Agena Bioscience, San Diego, CA, USA). The genotypes identified from MassARRAY analysis were cross-checked with the expected genotypes from the DArTseq data.

### Sexing markers

Four putative sex chromosome markers were previously identified for *M. gigas* in Ottewell et al.^38^. To improve efficiency of data handling and to reduce processing time, we developed TaqMan probes to sex scats on a real-time PCR (qPCR) machine. Multiplexed probes were designed using the PrimerQuest™ online tool (https://sg.idtdna.com/Primerquest/Home/Index).

The newly developed TaqMan probes and selected dyes are listed as followed:

- DDX3Y: 5′-/56-FAM/CGCCGTAAGCAATACCCAGTCTCC/3IABkFQ/-3′.
- SRY: 5′-/5HEX/TTTGCACCAGGAGAAATACCCGGA/3IABkFQ/-3′.
- Zfy: 5′-/5TEX615/TGTGCTATGGAACTCATGTGCCCT/3IAbRQSp/-3′.
- Zfx: 5′-/5Cy5/CCAAGGAAATCATTCATGAATATCA/3IAbRQSp/-3′.

We tested and optimised the new probes using one male tissue and three scats with three different concentrations of primer/probe mixes (0.5, 1.0, and 2.0 μM, Supplementary 2 Fig. S1). Then, we tested sex allocation consistency with eight tissue samples (four females and four males) and six scats (Supplementary 2 Fig. S2). Zfy was excluded in the second trial and subsequent runs because DDX3Y and SRY provided sufficient data. The final reaction consisted of primer and probe mix (1.0 μM) amplified in 10 µL reactions using the PrimeTime™ Gene Expression Master Mix (Cat No: 1055772) as per the manufacturer instructions with an annealing temperature of 60 °C, 40 amplification cycles and 4 µL of faecal DNA. The reactions were run on the CFX96™ Real-Time System C1000 Touch Thermal Cycler (BIO-RAD, Singapore) and analysed using the CFX Maestro software (BIO-RAD, Singapore).

### Case study

**Figure 1:**
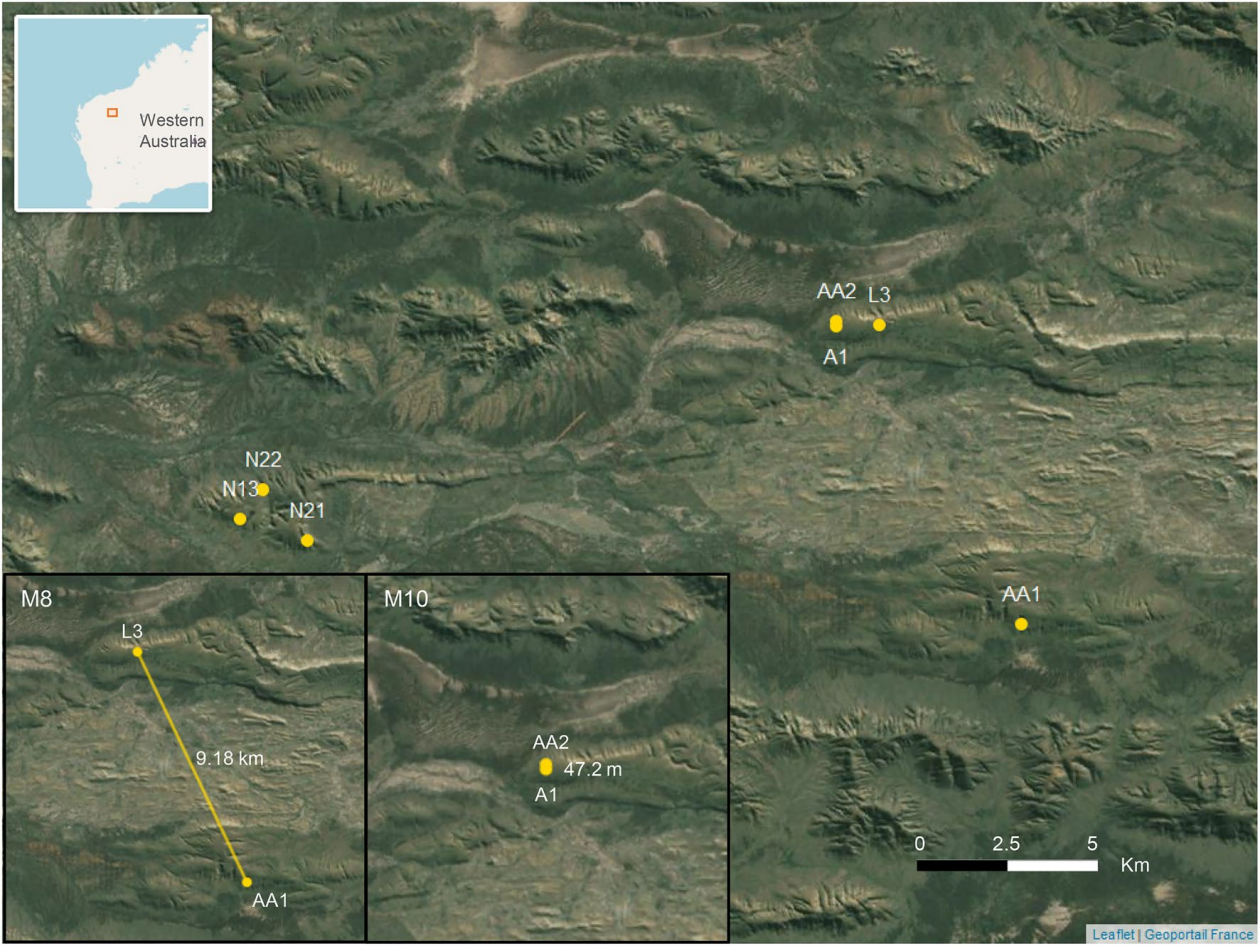
The spatial arrangement of roosts where *Macroderma gigas* scats were collected. The top insert is the site location within Western Australia and the bottom inserts are movement patterns of *M. gigas* male number 8 (M8) and male number 10 (M10). The map is generated by an R package ‘leaflet’ version 2.2.1^76^.

Scats (n = 209) were collected from seven roosts in West Angelas between 15–19 October 2019 (Fig. 1). At each roost, a maximum of 20 fresh to moderately fresh *M. gigas* scats were collected from tarpaulins placed on the cave floor for several months. We chose single scats that were not touching others to avoid any cross-contamination, and placed each into an individual envelope. Samples were kept frozen until DNA extraction and every scat was handled separately onwards using sterile laboratory and handling procedures. DNA was obtained from the scats by scraping the outer surface of frozen scats with a blade. Scraped material was processed using the Omega Biotek Mag-Bind Stool DNA 96 kit following the manufacturer’s instructions with a modification of using 50% diluted elution buffer in the final elution step to reduce EDTA inhibition for downstream analyses. Samples were eluted in 100 μL elution buffer.

We employed a two-step genotyping protocol. In the first step, we genotyped all samples with the first SNP panel (Panel 1, 47 SNPs) to identify unique individuals (P_ID_ analysis indicates 20 markers were needed to separate related individuals; “Results”). We were unable to obtain matched tissue and scat samples to evaluate the performance of the panel on invasively-collected vs non-invasive samples due to ethical considerations as the species is highly sensitive to disturbance. Instead, we re-genotyped 10% of samples (23 randomly selected samples) with Panel 1 to ensure consistency across runs and allow calculation of allelic dropout error rate. Rather than using the multi-tubes approach employed previously for microsatellite studies, we accounted for genotyping error estimates from re-genotyping in the clustering analysis (i.e. 2.5% from allelic dropout × 47 loci is 1.2 SNP). This approach is much cheaper than a multi-tubes approach and the data is handled based on scat quality. MassARRAY SNP results were processed in a custom R package ‘ScatMatch’^45^. ScatMatch contains several custom functions and visualisations for assessing genotype quality and clustering of samples based on their genotype dissimilarity. First, scat genotypes are ‘cleaned’ based on sample and locus amplification rates (typically 90% and 80%, respectively) to retain only high-quality data. Samples with low amplification rates (Supplementary 3 Fig. S1) are likely to be older and contain more errors^4,13,14^. ScatMatch then employs hierarchical cluster analysis using the R package ‘stats’ with the function ‘hclust’ and the method “average”^42^ to group scats based on the number of allele mismatches. Each scat genotype is assigned initially to its own cluster, and then the algorithm proceeds iteratively to join the two most similar clusters, and continues until only one cluster is left. The clustering is visualised as a dendrogram and we determined the cut height *h* (essentially the number of allele mismatches) at which to accept clustered samples are from the same individual. This threshold is decided based on inspection of several data visualisations. An elbow graph (‘elbow_plot’ function) shows the numbers of groups or putative individuals identified with increasing numbers of allele mismatches, and with the clustering threshold determined by the point at which the number of groups stabilises. We looked for a clear distinction in the number of SNP differences between grouped and ungrouped genotypes when the pairwise dissimilarity matrix is visualised as a heat map (‘heat_plot’ function). Lastly, we assessed the clustering threshold from the misassignment graph (‘misassign’ function). The graph plots the frequency of allelic differences within groups (SNP differences among scats from the same individual) and between groups (SNP differences between scats from different individuals) assuming the allelic frequency to follow a binomial distribution. Any allelic differences falling into the within group distribution are likely to be genotyping errors resulting from variation in DNA quality between scat samples from the same individual. Each selected value of *h* generates an overlap of the upper 0.995 percentile of the within group distribution and the lower 0.005 percentile of the between groups distribution. A greater degree of overlap means a higher probability of misassigning scats to individuals or would indicate the lack of SNP variation for individual identification.

In the second step, after scats had been assigned to individuals by ‘ScatMatch’, we selected one sample per bat with the best amplification rate to be genotyped with MassARRAY Panels 2 and 3 to obtain population genetic information. We also selected up to three random samples per bat to determine sex using the TaqMan qPCR assay. Because the amplification of sexing markers from scats is not always consistent due to variable sample quality, we established several criteria for assigning sex. Amplification was considered successful if the qPCR amplification RFU signal was ≥ 50. Samples were considered male if they met additional criteria as follows: a ratio of Y- to X-linked RFU signal > 0.1; and consistently assigned to the same sex in multiple samples. ‘Likely’ sex was defined when a group of samples with a small level of disagreement among markers and/or samples and the sex selected made up the majority of the result. ‘Undetermined’ was defined when a group of samples with amplification signal below 50 RFU from multiple markers or sex could not be assigned with confidence (Supplementary 3 Table S1).

Using all filtered loci from three panels, summary population genetic diversity statistics such as observed heterozygosity (Ho), gene diversity or observed heterozygosity (Hs), allelic richness (A) and inbreeding coefficient (F_IS_) were calculated from all bats in the Hierfstat package in R^42^. The contemporary effective population size (N_e_) was estimated in NeEstimator^46^ using the Linkage Disequilibrium method^46,47^. The parametric method was used to calculate the 95% confidence intervals of each N_e_ estimate^48^. Pairwise genetic relatedness between all bats was calculated using an R package ‘Related’^49^. We used the Ritland estimator as recommended by Attard et al.^50^ for SNP data that gives a value of 1.0 for identical twins or clones, 0.5 for parent–offspring or full-sib, and 0.25 and 0.125 for second and third-order relationships^51^.

### Ethical approval

No animals were directly involved during study and only data has been used. The unpublished DArTseq data of Pilbara *M. gigas* tissue samples were collected under scientific permits issued by the Western Australian Department of Biodiversity, Conservation and Attractions (SF002775, SF003138, SF005423, and SF005774).

## Results

### SNP selection

**Figure 2:**
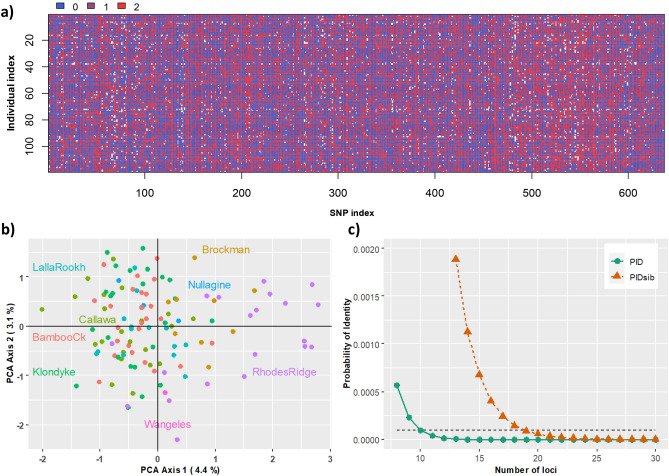
*Macroderma gigas* SNP selection for MassARRAY panels assessing for genotype variation for all 611 SNPs (**a**), A Pearson Principal Component Analysis of 150 SNPs (**b**), and Probability of Identity of 50 SNPs (**c**). Different colours in (**a**) represent 3 genotypes: blue (0) for a homozygote of the reference allele, purple (1) for a heterozygote, and red (2) for a homozygote of the alternative allele. Colours in (**b**) represent locations of *M. gigas* tissue samples collected across the Pilbara. Different P_ID_ colours in (**c**) represent the probability of identifying unrelated individuals (P_ID_, green) and related individuals (P_IDsib_, orange).

Out of 33,340 SNPs, 611 SNPs were identified as suitable, high quality, polymorphic candidate loci for the MassARRAY panels (Table 1). The filtered markers exhibited variation of homozygosity and heterozygosity in the in silico dataset as anticipated (Fig. 2a). A Pearson Principal Component Analysis of the original DArTseq genotypes showed an emergence of population structure pattern when more loci were included (Fig. 2b and Supplementary 3 Fig. S2). At the threshold of 0.0001, P_ID_ analysis of one panel (50 SNPs) showed that at least 10 or 20 loci were required to separate unrelated or related bats respectively (Fig. 2c). Inclusion of a larger number of loci did not change the result of P_ID_ analysis (Supplementary 3 Fig. S3), but it reduced standard deviations in population genetic diversity estimates although actual parameter estimates were consistent across all data subsets (Supplementary 3 Fig. S4). As a balance between per-sample genotyping costs and information content we designed three SNP panels. One MassARRAY SNP panel can contain up to 50 SNPs but due to constraints on multiplex primer design, our initial panels contained a total of 140 SNPs (Panel 1 = 47 SNPs, Panel 2 = 49, Panel 3 = 44, Supplementary 4).

### SNP panel performance

First, we compared genotyping success and error rates for seven tissue (20 ng/μL) and 11 scat (concentration unknown) samples. Based on 108 SNP loci with ≥ 50% amplification rate, tissue samples had an average sample amplification of 0.691 ± 0.082 (range 0.34–0.99) and 0.708 ± 0.067 (range 0.0–0.98) for scats. We allowed a lower amplification threshold because we used all three SNP panels for this trial. Average allelic dropout was low for both scat and tissue samples (tissue: 0.0 and scat: 0.038 ± 0.018). We also trialled the effects of DNA concentration on amplification and error rates. We found that the sample amplification rate ranged between 0.89 to 1.0 and the rate increased with DNA concentration but reached a plateau after 0.6 ng/μL (Supplementary 2 Fig. S3). Lastly, we compared sample amplification rates of scat DNA extracted with the Omega Biotek Mag-Bind Stool DNA 96 kit (Omega, USA, Cat No: M4016-01) and the QIAamp^®^ Fast DNA Stool Mini kit (Qiagen, Germany, Cat No: 51604). The average amplification rates, after removing completely failed samples, were also significantly higher for the Omega kit (0.901 ± 0.007, range 0.537–1.0) compared to the Qiagen kit (0.764 ± 0.013, range 0.293–1.0) (W = 20,605, P-value < 0.001, Supplementary 2 Fig. S4).

### Case study

**Figure 3:**
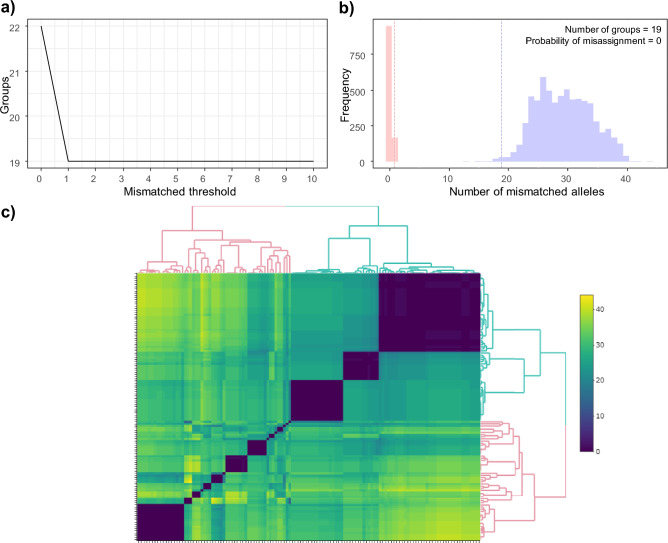
Threshold of allelic mismatch number to call *Macroderma gigas* scats from the same individuals with amplification rate above 90% for loci and 80% for scats. (**a**) Graph of different numbers of SNP mismatches allowed and numbers of group assigned to genotypes. (**b**) Misassignment graph (*h* = 1) demonstrates the frequency of pairwise allelic mismatches of raw genotypes where both samples have genotypes. The red and blue highlights represent SNP differences between faecal samples from ‘within’ and ‘between’ groups or putative individuals respectively. The red and blue dashed lines represent the upper 0.995 percentile and lower 0.005 percentile respectively. (**c**) Heatmap of pairwise allelic mismatches between all scats. Scats are clustered based on their similarity. A lower number of mismatches presents blue and a higher number of mismatches presents in yellow.

In the first step of our genotyping protocol, all samples were genotyped for Panel 1 to identify individuals represented in the collection of scats. Out of 209 scats and 23 replicates, 13 scats and 2 replicates (6.5%) failed to amplify. The overall sample amplification rate (excluding failed samples) ranged from 0.54 to 1.0, with a mean of 0.90 ± 0.01. Allelic dropout in repeated samples was 0.025 ± 0.004 and was negatively correlated with amplification rate (Supplementary 3 Fig. S1). Evaluation of alternative sample and locus filtering thresholds (Supplementary 3 Fig. S5) clearly shows the impact of data quality on the ability to confidently cluster scat genotypes. Removing lower quality samples/loci results in a clear plateau in the number of individuals identified regardless of the number of SNP mismatches past *h* = 1 visualised in the elbow graph, and greater distinction between ‘within individual’ (caused by genotyping errors) and ‘between individual’ (caused by biological variation) SNP differences (Supplementary 3 Fig. S5). As a result of these visualisations, we chose to filter our data to samples and loci with ≥ 0.9 and ≥ 0.8 amplification rates, respectively, which resulted in 125 scats and 40 loci (Panel 1), and allelic dropout rate reduced to 0.003 ± 0.000. For high quality samples, we identified the threshold SNP mismatch number as *h* = 1 based on inspection of elbow and misassignment graphs (Fig. 3a,b), with the heat map showing that scats tend to cluster together if there were SNP differences of ≤ 1 (Fig. 3c). Based on these analyses, we assigned scats with ≤ 1 SNP mismatch to the same bat.

**Figure 4:**
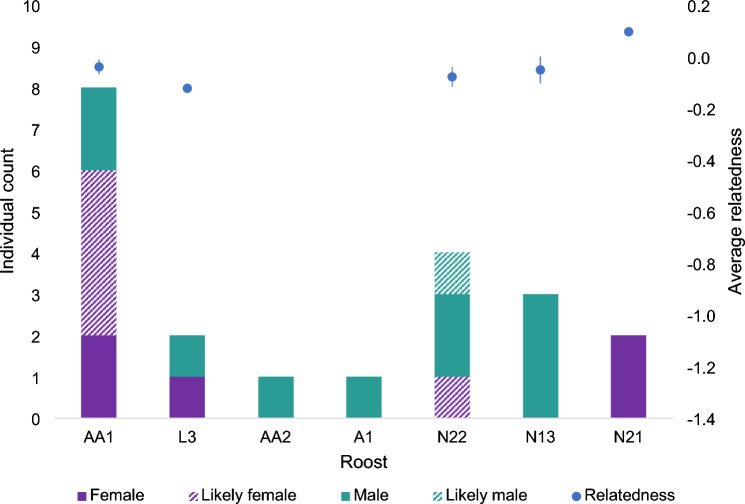
Summary of average pairwise genetic relatedness and sex of *Macroderma gigas* detected in each roost. Note that some bats used multiple roosts so they have been double-counted across these roosts. Likely sex is defined as a group of scats with some disagreement between markers and/or scats, but the sex selected made up the majority of the result. Bars around relatedness mean are standard error bars.

Based on stringent filtering criteria, we identified 19 unique genotypes (individuals) from 125 filtered scats and 40 filtered loci. Of the 19 individuals identified, there were eight males, one likely male, five females and five likely females (Supplementary 3 Table S2). This gave a sex ratio of males to females of 1.6:1 for confirmed sex, or 0.9:1 for all bats. We detected between 1 to 8 unique individuals per roost (Fig. 4). Stringent filtering resulted in a loss of 40% of the total number of scats, indicating 19 individuals as a minimum estimate of the number of bats present. Using more relaxed filtering criteria and threshold mismatch number allowed identification of further individuals (30 to 38 bats using loci/scat amplification rates of 0.8 and 0.7 respectively, Supplementary 3 Fig. S5), however, lowered data quality reduces confidence in these identifications.

Of 19 bats, we only detected two individuals (M8, M10) using more than one roost during the sampling period (Supplementary 3 Table S2) with the remaining bats only detected using a single roost. M8 was detected in roost AA1 and L3, 9.2 km apart while M10 was detected in roost AA2 and A1, 47 m apart (Fig. 1). Based on the number of scats, we infer that bats F_likely_5 (No. scats = 8), M6 (13), F7 (19), M8 (37), F9 (17), and M10 (7) spent significant time in the study area and were likely to be resident bats. While both M8 and M10 were detected in multiple roosts, based on the number of scats, M8 spent most of his time in roost AA1 while M10 spent a similar amount of time in both roosts (Supplementary 3 Table S2).

Roost AA1 had the largest number of scats and the highest number of bats detected (Fig. 4). Many of these bats were females or likely females, suggesting this cave may be a maternity roost. Interestingly, despite the likelihood of roost AA1 being used by breeding females, resident bats with many scats collected consisted of both sexes (Supplementary 3 Table S2). The remaining caves were clustered spatially, with only 3 bats detected using cluster L3/AA2/A1, including two resident bats F9 and M10. In contrast, 9 individuals were detected using cave cluster N22/N13/N21, however, only low numbers of scats were detected for each individual (n = 1–3) possibly indicating visits to these caves were occasional.

**Figure 5:**
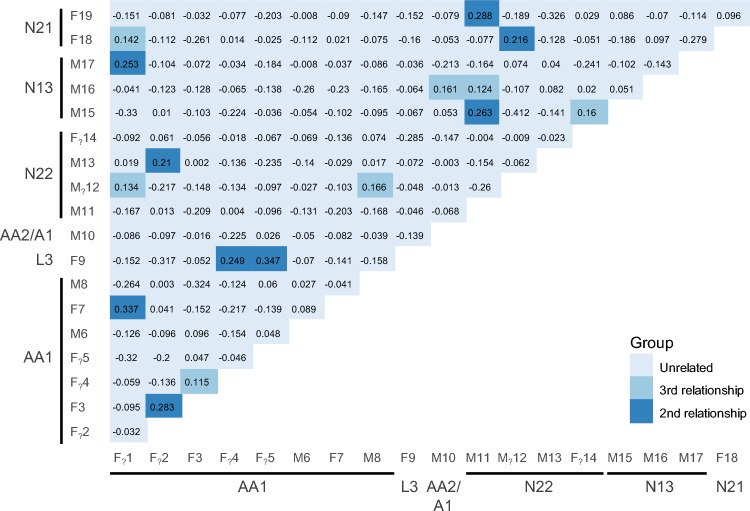
Pairwise genetic relatedness of *Macroderma gigas* calculated using Ritland^51^. The genotype IDs are sorted by roost IDs. Genotype IDs with ? indicates the likely sex. Colour intensity indicates genetic similarity in categories of first relationship (R = 0.5; parent–offspring, full-siblings), second relationship (R = 0.25; half-sibling, uncle/aunt–nephew/niece, grandparent–grandoffspring) and third relationship (R = 0.125; full cousin, great-grandparent–great-grandoffspring, great-uncle/aunt–great-nephew/niece, half-uncle/aunt–nephew/niece)^51^. No first relationship was detected.

Following identification of individual bats, representative scats from each individual were genotyped with all 93 loci on SNP Panels 2 and 3 to obtain population genetic diversity metrics. Loci were then filtered to those with ≥ 50% amplification rate (n = 74) before combined with loci from Panel 1 (n = 40) resulted in a total of 114 filtered SNPs. The group of bats detected in this site had an average observed heterozygosity of 0.40 ± 0.02 and expected heterozygosity 0.46 ± 0.01 indicating a moderate level of inbreeding (F_IS_ 0.14 ± 0.03). We anticipated that genetic relatedness may be higher in the maternity roost if there is maternal philopatry, implying that female offspring return to their natal cave to reproduce, as has been suggested in past population genetic studies^30,34^. Despite having more females in some roosts, average genetic relatedness was low in all roosts (overall mean R = − 0.04) and not significantly different between roosts (Fig. 4). All roosts consisted of unrelated individuals except one pair of bats from roost AA1 with a relatedness value closer to parent–offspring or full-sibling relationship and another similarly related pair located in AA1 and L3 (Fig. 5). The effective population size was estimated as 52.6 (CI 35.8–92.6).

## Discussion

Genetics-based non-invasive sampling is recognised as an efficient and cost-effective tool for monitoring rare and elusive species^52^. Mark-recapture approaches to estimate population abundance have relied traditionally on live capture, which risks injuries or death from capture and handling and may perform inadequately for species exhibiting trap avoidance behaviour^35^. The usefulness of faecal genotyping with microsatellite markers has been shown previously for addressing these issues for ad hoc monitoring of *M. gigas* populations^38,39^, however, the limited number of markers and higher error rates associated with this marker type suggest they may be problematic for use in statistical mark-recapture analyses. Transitioning from microsatellite markers to pre-selected SNP markers offers increased power to distinguish related individuals, improve faecal genotyping efficiency and reduce error rates. We developed and successfully tested 114 novel high-quality SNPs for automated genotyping of faecal DNA on the MassARRAY platform using a two-step genotyping protocol to firstly identify individuals from scats using a panel (‘Panel 1’) of 40 high-quality SNPs, and then estimating population genetic parameters (relatedness, genetic diversity and effective population size) from the total SNP set. Combining individual assignment and sexing information, we answered relevant demographic and ecological questions using scats collected across an 83 km^2^ study site in the eastern Pilbara. Below, we discuss the development of this technology in comparison to other studies, and in particular how the information gained from this technology provides insight into the biology of *M. gigas*. Lastly, we discuss limitations and possible future research directions of faecal genotyping.

### Performance of SNP panels for faecal DNA genotyping

Scat DNA analysis is often impacted by low DNA quality and quantity, which can lead to errors in assigning scats to individuals, and introduce biases into mark-recapture analyses^53^. We demonstrated that *M. gigas* scats produced a high locus amplification rate (90%) with very low genotyping error rates (< 2.5%), which was observed in both the trial and the case study at West Angelas. The average amplification and error rates in this study are comparable to other faecal genotyping studies using the MassARRAY platform (e.g. amplification rate 71%, allelic dropout or ADO rate 0–37% in Bornean elephant^54^; amplification rate 60%, ADO < 1% in pumas^55^). Low DNA concentration (< 0.6 ng/μL) negatively impacted the sample amplification rate in our trials, however we did not see a consistent pattern of increasing allelic dropout in our data, with low rates across all DNA concentrations tested (Supplementary 2). Microsatellite studies typically show a negative relationship between genotyping errors and amplification rate, with the rate of allelic dropout increasing with greater scat age (assumed to be a result of low DNA quantity and/or quality^4^). The better performance of *M. gigas* scats could be due to environmental factors since caves provide a stable microclimate where scats are sheltered from UV light and rain compared to those exposed to ambient weather conditions^4,5,16,17^. In addition, we found an interaction between the DNA extraction method used and MassARRAY amplification rate with the QIAamp^®^ Fast DNA Stool Mini kit performing more poorly than the Omega Biotek Mag-Bind Stool DNA 96 kit, most likely due to the presence of EDTA in buffers interfering with MassARRAY multiplex PCR reactions.

Overall, we found 94% of faecal samples in our empirical study produced genotypes, but after stringent filtering we retained only high-quality genotypes for analysis, representing a final success rate of 60%. Notwithstanding this relatively low rate, stringent filtering enabled us to match genotypes and identify individuals with a very high level of confidence (allowing 0–1 allelic mismatch between samples). Such high precision also builds trust in an ability to identify recaptures unambiguously within a mark-recapture framework. Allowing greater levels of missing data and a higher mismatch threshold between scats increased the number of scats available for analysis (81–89%) and doubled the numbers of individuals detected. However, relaxing data quality thresholds can increase misassignment of scats to individuals, either by failing to discriminate between scats from related individuals (overmerging) or by differentiating between scats from the same individual due to genotyping errors (oversplitting). Mark-recapture analysis relies on accurate identification of individuals to estimate recapture parameters; consequently misassignment of genotype IDs can have significant implications for estimates of population size made from such studies^56^.

Genotyping error rates have been reported in the range of < 1–49% in microsatellite studies^38,56,57^ but are typically lower for SNP arrays^18^. Pre-selection of SNP markers enables high performing bi-allelic loci to be targeted, and automated SNP allele calling on array platforms is less prone to genotype scoring error introduced by humans. The use of bi-allelic markers also reduces the impact of genotyping artefacts (‘false alleles’) that are often detected in microsatellite studies^58^. Improved control over the performance of genetic markers and consistency in genotype calling permit the increased confidence in identification of individuals for mark-recapture analysis. We also found that sexing markers were more sensitive to DNA degradation from a few inconsistent callings in scats assigned to the same individual. This could be due to null results or allelic dropouts, which were more frequently reported in fox faecal sexing markers than autosomal markers^15^.

Automated SNP genotyping is efficient for individual identification since pre-selection of highly variable SNP makers requires only a small number of markers to differentiate related individuals^18,52^. From simulated data, Glaubitz et al.^21^ showed that 22–28 biallelic loci are required to achieve an exclusion probability greater than 0.99 if these loci have a minimum allele frequency between 0.25 to 0.50. Similarly, here we found that 20 SNPs (frequency > 0.4) are required to differentiate related individuals (P_IDsib_ < 0.0001). Our simulations however showed that at least 100 SNPs are needed for reliable genetic diversity estimates and population structure analysis. A further limitation of using high frequency, highly variable SNP markers is, whilst cost-efficient, they will overestimate genetic diversity statistics and underestimate effective population size^59^. Such markers can be less sensitive to genetic diversity changes and hence may be less informative for conservation-related questions.

### Faecal genotyping reveals *M. gigas* biology

Our minimum estimate of 19 bats at the West Angelas study site is comparable to previous surveys at the same site where we detected 12 bats in 2015 and 24 bats in 2017 using microsatellite markers^60^. Consistent with previous observations of *M. gigas* across the Pilbara^31,60^, we detected low numbers of individuals per roost (1–8 bats per cave) with the largest number of bats detected in roost AA1 with a bias towards females (6F:2M) in this cave, suggesting it is likely be a maternity roost. Roost AA1 is one of the largest in the area and has characteristics of a maternity roost with a dome ceiling located at the back of the cave creating dark, warm and humid microclimates^31^. Breeding activity has also been previously reported at this site^31^. Despite a general expectation of female philopatry^30^, we detected only one close to first-order pedigree relationship (parent–offspring or sibling) pair in roost AA1. The average genetic relatedness was similar to other roosts and the incidence of first- and second-degree related individuals was not higher than in other roosts.

Two male bats were detected moving between roosts. Male 8 travelled 9 km between roosts AA1 and L3, but spent most of its time in roost AA1; while Male 10 only travelled between nearby caves AA2 and A1 that are 47 m apart, and appeared to use both roosts equally. Tracking studies of bats indicate movements of 10–15 km during foraging bouts^37^ or between diurnal roosts^61^, although Toop’s^35^ records of marked *M. gigas*, indicate longer dispersal distances between 20 and 50 km, with one bat travelling as far as 150 km, suggesting a high capacity for dispersal in this species. Likewise, recapture analyses from scats have detected inter-roost movements up to 36 km apart, with an average of 4.0–8.6 km^62–64^, although many bats are found to persist in the same roosts over multiple sampling periods, consistent with the observations in this study and with tracking studies showing bats consistently returning to the same diurnal roosts after foraging^37,61^.

Our genetic diversity estimates revealed a moderate level of inbreeding (F_IS_) for the population of *M. gigas* at West Angelas. Despite this observation, we found that mean relatedness within roosts was typically low (most individuals were unrelated) with only two individuals detected close to first-order pedigree relationships (parent–offspring or full-sib). Previous microsatellite analysis has indicated that genetic diversity in the Pilbara population of *M. gigas* is moderately high (Ho = 0.816), albeit lower than in colonies in the Northern Territory (Ho = 0.874–0.928; Worthington Wilmer et al.^30^). Further sampling of Pilbara roosts is required to place the population genetic diversity estimates from West Angelas obtained from SNP analysis into a regional context.

### Limitations and future directions

Mark-recapture analysis using non-invasive samples has been applied successfully to estimate colony size in bat species *Myotis sodalis*^65^, *Rhinolophus hipposideros*^66^, as well as to estimate annual changes in population size in iconic species such as the brown bear *Ursus arctos*^9^ and the Eurasian otter *Lutra lutra*^57^, demonstrating the viability of genetic mark-recapture approaches. Our single-session field survey case study demonstrated the ability of our novel SNP genotyping approach to confidently identify *M. gigas* individuals and their recapture using faecal DNA samples. Further monitoring sessions are required to establish a robust design statistical mark-recapture monitoring approach to estimate population abundance using a combination of closed and open sessions. Nevertheless, results from our single-session survey provided insight into aspects of *M. gigas* biology that can inform survey design and choice of population models in future studies, including confirmation of a high rate of cave fidelity and the spatial scale of inter-roost movements. Further, application of molecular sexing markers provides additional information that may enable application of sex-specific mark-recapture models, incorporating heterogeneity in recapture rates amongst sexes.

We designed our assay specifically for the Pilbara population of *M. gigas*, yet the species also persists in disjunct, threatened populations elsewhere in northern Australia. SNP panels are known to be impacted by ascertainment bias since SNP selection is typically made based on the allele frequencies of only a subset of individuals or populations that are available to study, rather than the global population^67,68^. Whilst we only had genomic data from eight colonies of *M. gigas*, these spanned the two main geographic sub-regions in the Pilbara (Chichester and Hamersley). We also have a priori knowledge of low genetic structure in *M. gigas* in the Pilbara^69^ suggesting our SNP panels can be effectively applied to non-sampled colonies in the Pilbara with potentially little or minor loss of utility. However, it is likely that our SNP panels will have lowered effectiveness if applied to disjunct populations elsewhere in Australia due to significant variation in allele frequencies amongst regions^30^. As a result, development of region-specific SNP panels may be necessary to apply this mark-recapture methodology elsewhere. Nevertheless, as a disturbance-sensitive Threatened bat species, faecal DNA monitoring, in combination with an appropriate survey design, provides an opportunity for obtaining robust population abundance information to assist in the conservation and management of the species.

Here, we applied stringent filtering criteria to obtain only high-quality samples and genotypes to ensure a high confidence in assigning scats to individuals. Previous reviews of non-invasive mark-recapture approaches^56,64^ have argued that stringently filtering genotype data could inadvertently bias individual recapture probabilities, violating mark-recapture models since individuals shed varying amounts of DNA^70^. Lukacs and Burnham^53^ suggest that allowing a small amount of genotyping error to account for heterogeneity in an individual’s capture probability^53^. In addition, acknowledging that genotyping errors are somewhat unavoidable in genetic mark-recapture studies, Lampa et al.^57^ recommended using specific population-models that account for misidentification error (e.g. Lukacs and Burnham^71^ estimator, α, implemented in the program MARK^72^).

As genomic technology advances, it is increasingly possible to obtain detailed population information on threatened species non-invasively. An exciting prospect in recent years is the use of DNA methylation changes as a biomarker to estimate chronological age in humans^73^ and non-model species, including the bat *Myotis bechsteinii*^74,75^. At this stage such approaches are only effective for tissue samples, however, it would be of interest to assess the application of such techniques to target DNA methylation from cells in the intestinal tract for scats. Adding markers for molecular ageing to our scat assays could provide further valuable information on the age structure of populations and recruitment rates to assist in conservation monitoring.

### Supplementary Information

Supplementary Information 1.

Supplementary Information 2.

Supplementary Information 3.

Supplementary Information 4.

## Data Availability

The datasets generated and/or analysed during the current study are available in the National Centre for Biotechnology Information (NCBI) Sequence Read Archive, accession number SRR25822243. Author K. Armstrong can be contacted for access to the unpublished raw DArTseq data.

## References

[1] Ceballos G (2015). Accelerated modern human-induced species losses: Entering the sixth mass extinction. Sci. Adv..

[2] Zemanova MA (2020). Towards more compassionate wildlife research through the 3Rs principles: Moving from invasive to non-invasive methods. Wildl. Biol..

[3] Carroll EL (2018). Genetic and genomic monitoring with minimally invasive sampling methods. Evol. Appl..

[4] Carpenter FM, Dziminski MA (2017). Breaking down scats: Degradation of DNA from greater bilby (*Macrotis lagotis*) faecal pellets. Austr. Mammal..

[5] Piggott MP (2004). Effect of sample age and season of collection on the reliability of microsatellite genotyping of faecal DNA. Wildl. Res..

[6] Lamb CT (2019). Genetic tagging in the Anthropocene: Scaling ecology from alleles to ecosystems. Ecol. Appl..

[7] Mengüllüoğlu D, Fickel J, Hofer H, Förster DW (2019). Non-invasive faecal sampling reveals spatial organization and improves measures of genetic diversity for the conservation assessment of territorial species: *Caucasian*
*lynx* as a case species. PLoS One.

[8] Nussberger B, Wandeler P, Weber D, Keller L (2014). Monitoring introgression in European wildcats in the Swiss Jura. Conserv. Genet..

[9] Norman AJ, Spong G (2015). Single nucleotide polymorphism-based dispersal estimates using noninvasive sampling. Ecol. Evol..

[10] Ford MJ (2011). Inferred paternity and male reproductive success in a killer whale (*Orcinus orca*) population. J. Hered..

[11] Ruegg KC (2014). Mapping migration in a songbird using high-resolution genetic markers. Mol. Ecol..

[12] Claramunt AMA (2018). Determination of the diet of the ghost bat (*Macroderma gigas*) in the Pilbara region of Western Australia from dried prey remains and DNA metabarcoding. Aust. J. Zool..

[13] Panasci M (2011). Evaluation of fecal DNA preservation techniques and effects of sample age and diet on genotyping success. J. Wildl. Manag..

[14] Skrbinšek T (2020). Effects of different environmental and sampling variables on the genotyping success in field-collected scat samples. Acta Biol. Slov..

[15] Berry O, Sarre SD, Farrington L, Aitken N (2007). Faecal DNA detection of invasive species: The case of feral foxes in Tasmania. Wildl. Res..

[16] Sittenthaler M (2020). Factors influencing genotyping success and genotyping error rate of Eurasian otter (*Lutra lutra*) faeces collected in temperate Central Europe. Eur. J. Wildl. Res..

[17] Nsubuga AM (2004). Factors affecting the amount of genomic DNA extracted from ape faeces and the identification of an improved sample storage method. Mol. Ecol..

[18] von Thaden A (2017). Assessing SNP genotyping of noninvasively collected wildlife samples using microfluidic arrays. Sci. Rep..

[19] Frantz AC (2003). Reliable microsatellite genotyping of the Eurasian badger (*Meles meles*) using faecal DNA. Mol. Ecol..

[20] Taberlet P (1996). Reliable genotyping of samples with very low DNA quantities using PCR. Nucleic Acids Res..

[21] Glaubitz JC, Rhodes OE, Dewoody JA (2003). Prospects for inferring pairwise relationships with single nucleotide polymorphisms. Mol. Ecol..

[22] Ekblom R (2021). Sample identification and pedigree reconstruction in Wolverine (*Gulo gulo*) using SNP genotyping of non-invasive samples. Conserv. Genet. Resour..

[23] Threatened Species Scientific Committee. Conservation advice *Macroderma gigas* ghost bat. (Department of the Environment and Energy, http://www.environment.gov.au/biodiversity/threatened/species/pubs/174-conservation-advice-05052016.pdf, 2016).

[24] WorthingtonWilmer J, Curtis KL (2012). Queensland's Threatened Animals.

[25] Hoyle SD, Pople AR, Toop GJ (2001). Mark–recapture may reveal more about ecology than about population trends: Demography of a threatened ghost bat (*Macroderma gigas*) population. Austral Ecol..

[26] Woinarski JCZ, Burbidge AA, Harrison PL (2014). The Action Plan for Australian Mammals 2012.

[27] Churchill SK, Helman PM (1990). Distribution of the Ghost Bat, *Macroderma gigas*, (Chiroptera: Megadermatidae) in central and South Australia. Aust. Mammal..

[28] Armstrong, K. N., Woinarski, J. C. Z., Hanrahan, N. M. & Burbidge, A. A. *Macroderma gigas* (amended version of 2019 assessment). *The IUCN Red List of Threatened Species 2021: e.T12590A209530568* (2021).

[29] Cramer VA (2022). Research priorities for the ghost bat (*Macroderma gigas*) in the Pilbara region of Western Australia. Aust. Mammal..

[30] Worthington Wilmer J, Moritz C, Hall L, Toop J, Pettigrew JD (1994). Extreme population structuring in the threatened ghost bat, Macroderma gigas: Evidence from mitochondrial DNA. Proc. R. Soc. Lond. Ser. B Biol. Sci..

[31] Armstrong KN, Anstee SD (2000). The ghost bat in the Pilbara: 100 years on. Aust. Mammal..

[32] Armstrong K (2010). Assessing the short-term effect of minerals exploration drilling on colonies of bats of conservation significance: A case study near Marble bar, Western Australia. J. R. Soc. West. Aust..

[33] Hanrahan N, Turbill C, Armstrong KN, Dalziell AH, Welbergen JA (2019). Ghost bats exhibit informative daily and seasonal temporal patterns in the production of social vocalisations. Aust. J. Zool..

[34] Worthington Wilmer J, Hall L, Barratt E, Moritz C (1999). Genetic structure and male-mediated gene flow in the Ghost Bat (*Macroderma gigas*). Evolution.

[35] Toop GJ (1985). Habitat requirements, survival strategies and ecology of the ghost bat, *Macroderma gigas*, Dobson (Microchiroptera, Megadermatidae) in central coastal Queensland. Macroderma.

[36] Kunz T, Fenton MB (2005). Bat Ecology.

[37] Augusteyn J, Hughes J, Armstrong G, Real K, Pacioni C (2018). Tracking and tracing central Queensland’s *Macroderma*—Determining the size of the Mount Etna ghost bat population and potential threats. Aust. Mammal..

[38] Ottewell K (2020). Development and optimisation of molecular assays for microsatellite genotyping and molecular sexing of non-invasive samples of the ghost bat, *Macroderma*
*gigas*. Mol. Biol. Rep..

[39] Spencer PBS, Tedeschi J (2016). An Initial Investigation into the Genetic Diversity, Structure and Short-Range Spatial-Use by Ghost Bat in the Hamersley Subregion of the Pilbara.

[40] dartR: Importing and analysing SNP and Silicodart Data Generated by Genome-Wide Restriction Fragment Analysis. https://CRAN.R-project.org/package=dartR (2019).

[41] Zheng X (2012). A High-performance computing toolset for relatedness and principal component analysis of SNP data. Bioinformatics.

[42] R Core Team. R: A language and environment for statistical computing. R Foundation for Statistical Computing (2020).

[43] Peakall R, Smouse PE (2012). GenAlEx 6.5: Genetic analysis in Excel. Population genetic software for teaching and research—An update. Bioinformatics.

[44] Waits LP, Luikart G, Taberlet P (2001). Estimating the probability of identity among genotypes in natural populations: Cautions and guidelines. Mol. Ecol..

[45] Huntley, B. ScatMatch: Functions to aid error filtering from SNP genotype data and determine group majorities. R package version 1.0.0 10.5281/zenodo.5091145 (2021).

[46] Do C (2014). NeEstimator v2: Re-implementation of software for the estimation of contemporary effective population size (Ne) from genetic data. Mol. Ecol. Resour..

[47] Waples RS (2016). A bias correction for estimates of effective population size based on linkage disequilibrium at unlinked gene loci. Conserv. Genet..

[48] Jones A, Ovenden J, Wang J (2016). Improved confidence intervals for the linkage disequilibrium method for estimating effective population size. Heredity.

[49] Lynch M, Ritland K (1999). Estimation of pairwise relatedness with molecular markers. Genetics.

[50] Attard CRM, Beheregaray LB, Möller LM (2018). Genotyping-by-sequencing for estimating relatedness in nonmodel organisms: Avoiding the trap of precise bias. Mol. Ecol. Resour..

[51] Ritland K (1996). Estimators for pairwise relatedness and individual inbreeding coefficients. Genet. Res..

[52] von Thaden A (2020). Applying genomic data in wildlife monitoring: Development guidelines for genotyping degraded samples with reduced single nucleotide polymorphism panels. Mol. Ecol. Resour..

[53] Lukacs PM, Burnham KP (2005). Review of capture–recapture methods applicable to noninvasive genetic sampling. Mol. Ecol..

[54] Goossens B (2016). Habitat fragmentation and genetic diversity in natural populations of the Bornean elephant: Implications for conservation. Biol. Conserv..

[55] Fitak RR, Naidu A, Thompson RW, Culver M (2015). A new panel of SNP markers for the individual identification of North American Pumas. J. Fish Wildl. Manag..

[56] Lampa S, Henle K, Klenke R, Hoehn M, Gruber B (2013). How to overcome genotyping errors in non-invasive genetic mark-recapture population size estimation—A review of available methods illustrated by a case study. J. Wildl. Manag..

[57] Lampa S, Mihoub J-B, Gruber B, Klenke R, Henle K (2015). Non-invasive genetic Mark-Recapture as a means to study population sizes and marking behaviour of the elusive Eurasian otter (*Lutra lutra*). PLoS One.

[58] Pompanon F, Bonin A, Bellemain E, Taberlet P (2005). Genotyping errors: Causes, consequences and solutions. Nat. Rev. Genet..

[59] Marandel F (2020). Estimating effective population size using RADseq: Effects of SNP selection and sample size. Ecol. Evol..

[60] Ottewell, K., McArthur, S., Leeuwen, S. V. & Byrne, M. Cave use by the Ghost bat (*Macroderma gigas*) at the West Angelas mine site. Final report to Biologic Pty Ltd. (Department of Biodiversity, Conservation and Attractions, 2018).

[61] Tidemann CR, Priddel DM, Nelson JE, Pettigrew JD (1985). Foraging behaviour of the Australian Ghost Bat, *Macroderma gigas* (Microchiroptera: Megadermatidae). Aust. J. Zool..

[62] Sun, R. & Ottewell, K. Cave use by the Ghost bat (*Macroderma gigas*) at Western Ridge mine site: SNP genotyping result corrected report 2020 (Department of Biodiversity, Conservation and Attractions, Kensington, 2021).

[63] Ottewell, K., Thavornkanlapachai, R., McArthur, S. & Byrne, M. Cave use by the Ghost bat (*Macroderma gigas*) in the Western Range mining precinct (Department of Biodiversity, Conservation and Attractions, Kensington, 2020).

[64] Sun, R., Ottewell, K. & McArthur, S. Cave use by the Ghost Bat (*Macroderma gigas*) in the Brockman mining precinct. Final report to Biologic Pty Ltd. (Department of Biodiversity, Conservation and Attractions, Kensington, 2021).

[65] Oyler-McCance SJ (2018). Genetic mark–recapture improves estimates of maternity colony size for Indiana bats. J. Fish Wildl. Manag..

[66] Puechmaille SJ, Petit EJ (2007). Empirical evaluation of non-invasive capture–mark–recapture estimation of population size based on a single sampling session. J. Appl. Ecol..

[67] Lachance J, Tishkoff S (2013). SNP ascertainment bias in population genetic analyses: Why it is important, and how to correct it. Bioessays.

[68] Malomane DK (2018). Efficiency of different strategies to mitigate ascertainment bias when using SNP panels in diversity studies. BMC Genomics.

[69] Ottewell K, McArthur S, Leeuwen SV, Byrne M (2017). Population genetics of the Ghost Bat (Macroderma gigas) in the Pilbara bioregion.

[70] Jansson L (2022). Individual shedder status and the origin of touch DNA. Forensic Sci. Int. Genet..

[71] Lukacs PM, Burnham KP (2005). Estimating population size from DNA-based closed Capture-Recapture data incorporating genotyping error. J. Wildl. Manag..

[72] White GC, Burnham KP (1999). Program MARK: Survival estimation from populations of marked animals. Bird Study.

[73] De Paoli-Iseppi R (2017). Measuring animal age with DNA methylation: From humans to wild animals. Front. Genet..

[74] Polanowski AM, Robbins J, Chandler D, Jarman SN (2014). Epigenetic estimation of age in humpback whales. Mol. Ecol. Resour..

[75] Wright PGR (2018). Application of a novel molecular method to age free-living wild Bechstein’s bats. Mol. Ecol. Resour..

[76] leafletR: Interactive web-maps based on the Leaflet JavaScript library (R package version 0.4-0, 2016).

