# Supplementary material for: Species-specific SNP arrays for non-invasive genetic monitoring of a vulnerable bat
Source: Sci Rep. 2024 Jan 22;14:1847. doi: 10.1038/s41598-024-51461-5 (PMC10803360; doi:10.1038/s41598-024-51461-5)
Supplement: Supplementary file 2 — Supplementary Information 2. [file 41598_2024_51461_MOESM2_ESM.docx]

# Supplementary 2 Method Validation

## Testing sexing markers with TaqMan probes

To test the newly developed TaqMan probes with the pre-designed sexing primers ^1^, we used 1 male tissue sample (1 ng/μL) and 3 unknown sex and DNA concentration scat samples. All primers and probes (DDX3Y, SRY, Zfy and Zfx) were amplified in 10 μL reactions using the PrimeTime® Gene Expression Master Mix (Cat No: 1055772) as per the manufacturer’s instructions, with an annealing temperature of 60°C, 40 cycles, 1 μL of template DNA and primers and probes mix to the final concentrations of 0.5 μM, 1 μM and 2 μM on Bio-RAD CFX96 Touch Real-Time PCR Detection System (Foster City, California).

The results showed all samples, regardless of sample types, amplified in all markers (Fig. S1). There was a positive correlation between primer concentration and RFU signals (Spearman rho range 0.90 – 0.96, P-value < 0.001 in all primer pairs). Based on the amplification of all primers, the scat samples are likely to come from males.

**Figure S1**. Relative fluorescence units (RFU) of the ghost bat sexing markers’ PCR product at different primers and probes concentration. X-axis represents RFU of Zfx marker while Y-axis represents RFU of DDX3Y, SRY and Zfy markers.

To test sexing allocation consistency, we used 8 (4 females, 4 males) tissue samples (1 ng/μL) and 6 scats (unknown sex and DNA concentration). All of these samples are from different individuals to the previous trial. Due to the amplification success of all primers, we removed Zfy from the multiplex to reduce cost. The reaction was carried out as described in the previous trial with the final primers and probes mix of 1 μM and DNA from scat samples increased to 4 μL. Each sample was genotyped twice. Samples were considered male if amplification was successful for at least one Y-linked markers with the RFU threshold ≥ 50 and consistent between 2 replicates.

The results showed a consistent allocation of sex for the tissue samples (Fig. S2). Both replicates of two female samples showed DDX3Y amplification signals above 50 RFU, but the ratio of Y RFU to X RFU were low and ranged from 0.0 to 0.10 compared to males which ranged from 0.68 to 0.76. Based on these results, the ratio of DDX3Y to Zfx RFUs should be considered when allocating sex to scat samples and a minimum ratio of 0.1 should be applied. SRY amplification in female samples showed none to very low amplification signals and the ratio of Y RFU to X RFU in males ranging 0.33 to 0.40. This suggests that a minimum ratio of 0.1 should be applied to be consistent for both sets of markers. Scat samples produced much lower RFU signals compared to tissue samples. Based on the observation of tissue samples, we detected 3 males, 1 female and 2 undetermined due to poor amplification from scat samples.

**Figure S2.** Relative fluorescence units (RFU) of the ghost bat sexing markers’ PCR product, Zfx, DDX3Y and SRY with TaqMan probes. A is a comparison between Zfx (X-linked) and DDX3Y (Y-linked). B is a comparison between Zfx (X-linked) and SRY (Y-linked). Blue, red and green markers represent male tissue, female tissue and unknown sex scat samples respectively.

## Tissue and scat genotyping success and error rate

To test sample type performance, we compared 7 tissue samples (20 ng/μL) and 11 scat samples (unknown DNA concentrations). Tissue samples were DNA extracted using the salting out protocol ^2^ and scat samples were DNA extracted using the QIAamp® Fast DNA Stool Mini kit (Qiagen, Hilden, Germany) as described in Ottewell, et al. ^1^. We re-genotyped six scat samples twice to three times and five tissues twice (20 ng/μL) to estimate genotyping consistency and allelic dropout rate.

Based on 108 SNP loci with ≥ 50% amplification rate, tissue samples had an average amplification of 69.1 ± 8.2% (range 34% – 99%) for tissues and 70.8 ± 6.7% (range 0% – 98%) for scats. Average allele dropout was low for both scat and tissue samples (scat: 3.8 ± 1.8% and tissue: 0%).

## Effects of different DNA concentrations on amplification and error rates

To test effect of DNA concentration on genotyping success and allelic dropout rates, four DNA tissue samples were diluted to 0.1, 0.2, 0.6, 0.8, and 1 ng/μL because sending them to AGRF for genotyping all three SNP panels on the MassARRAY system. Allelic dropout rates were calculated against the common genotype derived from all samples at different DNA concentrations genotyped from the same tissue sample. Allelic dropout is counted when the consensus genotype at a particular locus is a heterozygote, but the sample at that locus showed only one out of two alleles.

Two samples, one at 0.6 ng/μL and another at 1.0 ng/μL failed completely and were removed from further calculation. Twenty four of 134 loci were removed from the analysis because 22 loci failed completely, and 2 loci had > 50% fail rate. One out of 4 samples at 0.8 ng/μL was removed because the amplification rate was much lower than other samples and was likely to be an outlier. Based on the remaining samples and loci, the sample amplification rate ranged between 0.89 to 1.0. Overall, average DNA concentration of all treatments were above 90% (Fig. S3). Amplification rate increased with DNA concentration but reached a plateau after 0.6 ng/μL (Fig. S3). All samples regardless of their concentrations matched the consensus genotypes except for one locus in one sample which showed a homozygote of alternate allele and another sample showed one locus with allelic dropout. Thus, the allelic dropout rates were zero on average across all concentrations.

**Figure S3**. Average amplification rates of 110 SNPs (≥ 50% amplification rate) genotyped from the designed three MassARRAY panels using four ghost bat DNA tissue samples diluted at different concentrations. Error bars are standard errors.

## DNA extraction kits comparison

To demonstrate the effectiveness of different DNA extraction kits on the amplification rate, we compared the Omega Biotek Mag-Bind Stool DNA 96 kit (Omega, USA, Cat No: M4016-01) to QIAamp® Fast DNA Stool Mini kit (Qiagen, Germany, Cat No: 51604). The Omega kit was used to extract samples from this manuscript. The Qiagen kit was used to extract samples used in Ottewell, et al. ^1^. No replicates were included in this analysis.

We found that the Omega kit performs better than the Qiagen kit. The proportion of samples with zero amplification was lower in the Omega kit (6.2%) compared to the Qiagen kit (15.6%) (Fig. S4). The average amplification rates, after removing completely failed samples, were also significantly higher for the Omega kit (0.901 ± 0.007, range 0.537 – 1.0) compared to the Qiagen kit (0.764 ± 0.013, range 0.293 – 1.0) (W = 20605, P-value < 0.001, Fig. S4).

**Figure S4**. Boxplots of ghost bat faecal sample amplification rates extracted with the Omega Biotek Mag-Bind Stool DNA kit and QIAamp® Fast DNA Stool Mini kit. One dot represents one sample.

The difference in performance could due to the presence of EDTA in the elution buffer of the Qiagen kit and the efficiency of different kits. At a high concentration, EDTA can act as a PCR inhibitor by depleting magnesium ions ^3^. The Qiagen kit had been used for ghost bat scat genotyping previously with microsatellite markers ^1,4^. The presence of EDTA in the elution buffer did not seem to interfere with PCR reaction. However, we found that MassARRAY is more sensitive to EDTA. In an earlier trial, we found that by diluting DNA samples can improve the amplification rate. Nonetheless, the improvement was not as high as changing the DNA extraction kit. Another possible reason for differences in performance is the efficiency of the DNA extraction kit. Husakova, et al. ^5^ found that certain commercial DNA extraction kits performed better than others for different types of source DNA regardless of the filtering method.

**References**

1 Ottewell, K. *et al.* Development and optimisation of molecular assays for microsatellite genotyping and molecular sexing of non-invasive samples of the ghost bat, *Macroderma gigas*. *Molecular Biology Reports* **47**, 5635-5641, doi:10.1007/s11033-020-05544-x (2020).

2 Sunnucks, P. & Hales, D. F. Numerous transposed sequences of mitochondrial cytochrome oxidase I-II in aphids of the genus Sitobion (Hemiptera: Aphididae). *Molecular Biology and Evolution* **13**, 510-524, doi:10.1093/oxfordjournals.molbev.a025612 (1996).

3 Schrader, C., Schielke, A., Ellerbroek, L. & Johne, R. PCR inhibitors – occurrence, properties and removal. *Journal of Applied Microbiology* **113**, 1014-1026, doi:<https://doi.org/10.1111/j.1365-2672.2012.05384.x> (2012).

4 Ottewell, K., McArthur, S., Leeuwen, S. V. & Byrne, M. Population genetics of the Ghost Bat (*Macroderma gigas*) in the Pilbara bioregion. Final report to Biologic Pty Ltd., (Department of Biodiversity, Conservation and Attractions, Kensington, Western Australia, 2017).

5 Husakova, M., Kralik, P., Babak, V. & Slana, I. Efficiency of DNA isolation methods based on silica columns and magnetic separation tested for the detection of *Mycobacterium avium* subsp. *Paratuberculosis* in milk and faeces. *Materials* **13**, 5112, doi:10.3390/ma13225112 (2020).
