# Supplementary material for: Species-specific SNP arrays for non-invasive genetic monitoring of a vulnerable bat
Source: Sci Rep. 2024 Jan 22;14:1847. doi: 10.1038/s41598-024-51461-5 (PMC10803360; doi:10.1038/s41598-024-51461-5)
Supplement: Supplementary file 3 — Supplementary Information 3. [file 41598_2024_51461_MOESM3_ESM.docx]

# Supplementary 3 SNP data handling

#

**Figure S1**. Spearman rank correlations between scat amplification and allelic dropout rate calculated from 21 replicate *Macroderma gigas* scats.

**Figure S2**. The Pearson Principal Component Analysis of different *in silico* SNPs numbers (insert top left). Analyses based on DArTseq data from eight locations in the Pilbara region indicated in different colours.

**Figure S3**. Probability of Identity of *in silico* 50, 100, 150 and 200 SNPs analysed from *Macroderma gigas* DArTseq data. Different P_ID_ colours represent the probability of identifying unrelated individuals (P_ID_, green) and related individuals (P_IDsib_, orange).

**Figure S4**. Genetic diversity estimates by different loci numbers from *Macroderma gigas* tissue samples collected from six locations (n > 8) in the Pilbara. Ho, Hs and Fis represent observed heterozygosity, gene diversity and inbreeding coefficient respectively. A bar around the mean is a standard deviation from 500 permutations.

**Figure S5**. Threshold of SNP mismatch number to call scats from the same individual (**a**) and amplification filtering threshold comparisons (**b**) for scat and loci amplification rates. The filtering used is indicated on the top right and the filter setting for the case study is shown in the last panels. The number of mismatch is calculated from pairwise comparison between scats with genotyping scores. Assuming the allelic frequency in b) follows a binomial distribution, allele mismatches forming the main peak on the right are likely to be the biological variations between individuals while allele mismatches on the left are likely to be variations between scat samples from the same individual.

**Table S1**. Raw sexing array results of SRY, DDX3Y, and Zfx markers from qPCR. Red, yellow and green colours indicate replicate samples, samples with < 50 RFU, and samples with a ratio of Y- to X-linked > 0.1.


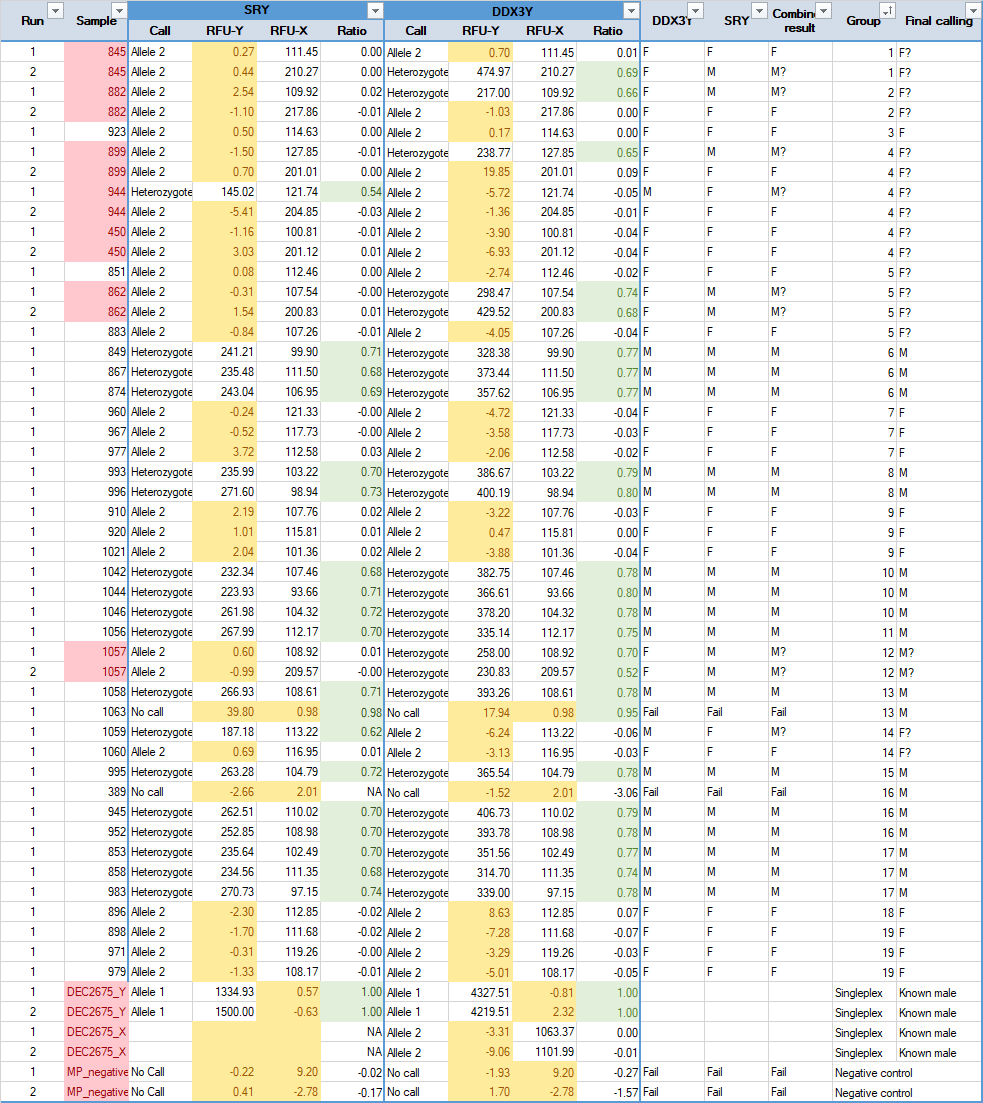


**Table S2**. Detection of *Macroderma gigas* in the West Angelas studied site by faecal scat numbers and roost visitations between 15 – 19 October 2019. F, M and ? indicate females, males and likely sex respectively.

| **Roost** | **Sex / Individual number** | | | | | | | | | | | | | | | | | | | **Bat total** |
| --- | --- | --- | --- | --- | --- | --- | --- | --- | --- | --- | --- | --- | --- | --- | --- | --- | --- | --- | --- | --- |
|  | **F?** | **F?** | **F** | **F?** | **F?** | **M** | **F** | **M** | **F** | **M** | **M** | **M?** | **M** | **F?** | **M** | **M** | **M** | **F** | **F** |  |
|  | **1** | **2** | **3** | **4** | **5** | **6** | **7** | **8** | **9** | **10** | **11** | **12** | **13** | **14** | **15** | **16** | **17** | **18** | **19** |  |
| AA1 | 1 | 1 | 1 | 4 | 8 | 13 | 19 | 36 |  |  |  |  |  |  |  |  |  |  |  | 8 |
| L3 |  |  |  |  |  |  |  | 1 | 17 |  |  |  |  |  |  |  |  |  |  | 2 |
| AA2 |  |  |  |  |  |  |  |  |  | 3 |  |  |  |  |  |  |  |  |  | 1 |
| A1 |  |  |  |  |  |  |  |  |  | 4 |  |  |  |  |  |  |  |  |  | 1 |
| N22 |  |  |  |  |  |  |  |  |  |  | 1 | 1 | 2 | 2 |  |  |  |  |  | 4 |
| N13 |  |  |  |  |  |  |  |  |  |  |  |  |  |  | 1 | 3 | 3 |  |  | 3 |
| N21 |  |  |  |  |  |  |  |  |  |  |  |  |  |  |  |  |  | 1 | 3 | 2 |
| Scat total | 1 | 1 | 1 | 4 | 8 | 13 | 19 | 37 | 17 | 7 | 1 | 1 | 2 | 2 | 1 | 3 | 3 | 1 | 3 |  |
